# Young adult preference analysis on the attributes of COVID-19 vaccine in the Philippines: A conjoint analysis approach

**DOI:** 10.1016/j.puhip.2022.100300

**Published:** 2022-07-19

**Authors:** Ardvin Kester S. Ong, Yogi Tri Prasetyo, Fae Coleen Lagura, Rochelle Nicole Ramos, Jose Ma Luis Salazar, Keenan Mark Sigua, Jomy Anne Villas, Thanatorn Chuenyindee, Reny Nadlifatin, Satria Fadil Persada, Kriengkrai Thana

**Affiliations:** a School of Industrial Engineering and Engineering Management, Mapúa University, 658 Muralla St., Intramuros, Manila, 1002, Philippines; b Department of Industrial Engineering and Management, Yuan Ze University, 135 Yuan-Tung Road, Chung-Li, 32003, Taiwan; c Young Innovators Research Center, Mapúa University, 658 Muralla, St., Intramuros, Manila, 1002, Philippines; d School of Graduate Studies, Mapúa University, 658 Muralla St., Intramuros, Manila, 1002, Philippines; e Department of Industrial Engineering and Aviation Management, Navaminda Kasatriyadhiraj Royal Air Force Academy, Bangkok, 10220, Thailand; f Department of Information Systems, Institut Teknologi Sepuluh Nopember, Kampus ITS Sukolilo, Surabaya, 60111, Indonesia; g Entrepreneurship Department, BINUS Business School Undergraduate Program, Bina Nusantara University, Jakarta, 11480, Indonesia

**Keywords:** COVID-19 vaccine, Filipinos, Orthogonal design

## Abstract

**Objective:**

Vaccines are utilized to prevent the severity of illnesses like the COVID-19 virus. Currently, there are a lot of COVID-19 vaccines available in the market like Pfizer, Moderna, AstraZeneca, Johnson and Johnson, and Sinovac. This research aimed to analyze the preference on the existing vaccine attributes of COVID-19.

**Study design:**

Specifically, this study considered 7 attributes such as cost, brand, recommendations, efficacy, side effects, vaccine type, and dose.

**Methods:**

A conjoint analysis with orthogonal design was utilized and 865 respondents were participated.

**Results:**

The result showed that consumers considered brand as the highest attribute, specifically Pfizer and Moderna among other brands. Moreover, the efficacy of 90% and higher were the preferred vaccine with 1 in 100 patient side effects reported. It was seen that safety and effectiveness is the priority in choosing a COVID-19 vaccine. Interestingly, the knowledge and understanding of the COVID-19 vaccine was found to drive consumer's preference for the vaccines available.

**Conclusions:**

The findings of this study could be utilized by the government to increase the willingness to be vaccinated. Lastly, the result of this study would pave a way to promote herd immunity to help fight the COVID-19 pandemic worldwide.

## 1 Introduction

Vaccines are substances injected into the body to help prepare antibodies to fight off different viruses [1]. The World Health Organization (WHO) stated that vaccines help in preventing the person to be severely ill by introducing weakened or sequenced viruses to the body [2]. Last April 2020, WHO evaluated vaccines that were distributed across the world for the COVID-19 virus [3]. With the current COVID-19 pandemic, at least seven different vaccines have been tested, accepted, and prescribed as effective [4]. Based on the Centers for Disease Control and Prevention (CDC), all the vaccines being developed underwent evaluations and clinical trials to prove its effectiveness. With the evaluated vaccines, only 4 met the WHOs’ criteria for safety and efficacy namely: AstraZeneca/Oxford vaccine, Johnson and Johnson, Moderna, and Pfizer/BionTech [1]. Despite the advice to be vaccinated, people tend to have different perceptions about being vaccinated [5]. Several factors such as effectiveness and affordability may affect the choice of people to accept the vaccine [6,7].

Aside from the lack of a more aggressive vaccine rollout, a high number of new cases daily, as well as other issues regarding the COVID-19 pandemic, consumers also show concerns regarding the fast development of the COVID-19 vaccines [[8], [9], [10], [11]]. The collection of these issues prompted consumers to have high particularity with regards to vaccine preferences. The preference of the COVID-19 vaccines being developed abroad is a major influence [9,10]. Many consumers expressed preferences for Pfizer, AstraZeneca, Johnson & Johnson, Moderna, and Novavax, which are all Western COVID-19 vaccines. In addition, Filipinos are least considering vaccines from China [9,10]. Aside from the brand, factors that may affect the preference of vaccines may be cots and efficacy [12,13].

It is well established by several studies that vaccines will lead to individual immunity against diseases and protect people with no ability to get vaccines through herd immunity [7,[14], [15], [16]]. However, a study conducted by McKee and Bohannon [17] suggested that there are parents who refuse or delay vaccination for their children. Interestingly, the World Health Organization perceives vaccine hesitancy as a top threat to public health [18]. As such, there have been many studies conducted determining vaccine choices and preferences of individuals, especially adult women for their children [19]. One example is a study conducted by Sun et al. [20] suggests that it is imperative for vaccine promotion in China to focus on parent's stated preferences including prior testing to Chinese children. Additionally, Wong et al. [16] found that governmental recommendation is a significant driving factor with acceptance of the COVID-19 vaccine. In this light, Seanehia et al. [21] suggested that conjoint analysis must be conducted to explore vaccine hesitancy in particular vaccination programs.

Grounded in conjoint measurement theory, conjoint analysis is a tool to measure the significance of preferences by highlighting certain attributes [[21], [22], [23], [24], [25], [26]]. Health care researchers have widely utilized the use of conjoint analysis such as food choices [[27], [28], [29], [30], [31]], patient treatment preferences [[32], [33], [34]], and even HIV medications [35,36]. There are also studies that utilized conjoint analysis regarding vaccines similar to this study. Sun et al. [20] did a study focusing on stated vaccine preferences specifically in China. Seanehia et al. [21] also did a study on vaccination against rare diseases by quantifying population preferences among French university students. Similarly, Stockwell et al. [19] focused on adult women's attitudes on vaccination and the effects of vaccine characteristics. The conjoint analysis studies aforementioned are focusing on vaccines, however, does not focus on the COVID-19 vaccines. COVID-19 vaccines are new kinds of vaccines and are currently underexplored. Taking into account the country, there has been no studies conducted with regards to the preference among adolescents in the vaccine uptake. Recent news from the CDC has recommended vaccination uptake for 12 years old and above. Thus, using a conjoint analysis approach using orthogonal design in determining COVID-19 vaccine preferences would be viable. This would help in lessening the spread of the COVID-19 virus as the vaccination uptake may increase when preference is being catered.

This research aimed to analyze the preference on the existing vaccine attributes of COVID-19. Specifically, conjoint analysis with the orthogonal design was utilized to analyze 7 attributes such as cost, recommendations, brand, efficacy, side effect, vaccine type, and doses. Furthermore, this study is the first study that considered young Filipino adults using conjoint analysis to analyze the preferences among the existing COVID-19 vaccines in the Philippines. The result of this study may be beneficial for herd immunization since the preference of the citizens were considered. In addition, the results of this study can pave a way to convince and introduce mass vaccination in a country. Lastly, the analysis of this study can be applied to analyze the preferences among the dynamic changes and developments of the COVID-19 vaccinal attributes in a local or international setting.

## 2 Methodology

### 2.1 Participants

The researchers conducted an online survey to determine the preference to get the COVID-19 vaccine. Convenience sampling was utilized as the sampling criterion to ensure that the probability of being chosen is equal among the population. The average time of completing the questionnaire was 20 min together with the consent form. This study was approved by Mapua University Research Ethics Committees and followed the National Ethical Guidelines for Health and Health-Related Research 2017 by the Philippine Health Research Ethics board.

The researchers obtained 865 valid responses which provided sufficient data needed for this research. The survey was distributed by utilizing social media platforms due to the current situation of the COVID-19 pandemic in the country. Moreover, the data was collected from October 2021–November 2021 when the COVID-19 vaccine uptake for 12 years old and above was announced. The Philippines declared the uptake to start from November of 2021 and thus the consideration of data collection prior to vaccine uptake. As stated in the study of Sethuraman et al. [37], an online survey distributed for conjoint analysis is viable.

### 2.2 Conjoint design

Table 1 represents the attributes and levels considered in this study. There was a total of 7 attributes considered namely: cost, recommendations, brand, efficacy, side effect, vaccine type, and doses.

**Table 1:** Attributes for COVID-19 preference.

Attributes	Level
Brand	Pfizer
	Sinovac
	AstraZeneca
	Johnson and Johnson
	Moderna
Recommendation	World Health Organization
	Personal Physician
	Personal Preference
Cost	Free from the government
	Discounted (Government discount)
	Fully paid
	Free from employer
Efficacy	95%
	50.4%
	76%
	66.3%
	94%
Side Effects	1 in 2 patients
	1 in 50 patients
	1 in 100 patients
Vaccine Type	mRNA
	Weakened virus
Doses	1
	2

Potential COVID-19 vaccines may vary from different brands that consequently affects COVID-19 vaccine acceptance. As such, the conjoint experimental design in this study includes brands as its first attribute with 5 levels. These levels were composed of different brands which are Pfizer, Sinovac, AstraZeneca, Johnson and Johnson, and Moderna. Perhaps relatedly, different brands that manufacture COVID-19 vaccines come from different countries around the world. According to Motta [15], the origin of a vaccine affects vaccine candidates. For example, there was widespread misinformation that stated that China plays a crucial role in creating the virus [38]. According to Laughlin and Shelburn [39], it may be a possible consequence that survey findings suggest that Americans prefer vaccine brands in the U.S. compared to China.

Secondly, vaccine candidates have the potential to vary from recommendations. According to Kreps and Kriner [40] safety concerns and lack of trust of doctors who recommend vaccines are often said as the cause of vaccine hesitancy. Subsequently, the conjoint experimental design included three levels for the recommendation attribute which are: (1) WHO, (2) personal physician, and (3) personal preference. Zandian et al. [41] even recommended that knowledge when it comes to the COVID-19 from practitioners and even the ministry promotes positive behavior among students. The reason for this circumstance roots in the fact that political or organizational endorsement on vaccine acceptance has become a significant factor [40].

The third attribute that was included in the cost which the vaccine candidates will vary. According to Connochie et al. [42], monetary costs have been integrated into measures by individuals inspecting vaccine acceptability. However, it is found to have small effects on vaccine acceptability compared to efficacy rate. Nevertheless, 1% of 2400 Filipinos respondents conducted by Pulse Asia have said that they have vaccine hesitancy due to vaccine candidates' potential unavailability to be free; while another 1% have vaccine hesitancy that roots from the vaccine candidates' monetary expensiveness [12]. As such, the conjoint experimental design's third attribute includes 4 levels which are: (1) free from the government, (2) Discounted (Government discount), (3) Fully Paid, and (4) Free from the employer.

Fourth, vaccine candidates will differentiate depending on their potential efficacy rates that determine their safety and effectiveness at fighting the COVID-19 virus. As such, the conjoint experimental design includes efficacy as an attribute. Moreover, levels of the efficacy attribute include: (1) 95%, (2) 50.4%, (3) 76%, (4) 66.3%, and (5) 94%. These levels were based on the aforementioned brands, with the set minimum 50% effectiveness threshold to be disseminated by the Food and Drug Administration (FDA) and effectiveness of greater than 90% for leading vaccine candidates as revealed by early late-stage clinical trial data [15]. Consequently, it is surmised that preference on a COVID-19 vaccine with a higher efficacy rate than those that are less effective could be part of the preference measurement.

The fifth attribute that was considered is the Side Effects. According to Riad et al. [43], the Side Effects of COVID-19 vaccines have a vital role in the public's confidence toward the vaccine and vaccination intention. In addition, concerns about side effects are a factor in drawing negative perceptions [44]. However, it is important to note that proper understanding can draw a positive attitude towards the vaccine [45]. Thus, this study considered the frequency of side effects related to vaccination to be able to assess the consequence of side effects in the perceptions of an individual towards the current COVID-19 vaccines. The frequency that was considered is 1 in 2 patients (Very common), 1 in 50 patients (Frequent), and 1 in 100 patients (Infrequent) [46].

Sixth, this study also considered vaccine type as an attribute. The Centers for Disease Control and Prevention [4] stated that the current COVID-19 vaccines consist of two types: COVID-19 mRNA Vaccines and Viral Vector COVID-19 Vaccines (Weakened Virus). Viral Vector Vaccine is a type of vaccine that uses a different type of safe virus to deliver proteins that can trigger immunity without causing the disease [47]. Similarly, mRNA vaccine is a type of vaccine that instructs cells to generate proteins that can trigger an immune response [4]. Moreover, it is important to assess the patient's vaccination status, determine the type of vaccine needed for a patient, and assess for contradictions and precautions to ensure safe and effective vaccination [48].

The last attribute that was considered is the number of doses. Currently, Some of the COVID-19 vaccines require a single dose, while some require two [47,[49], [50], [51], [52]]. According to Cleveland Clinic [52], the number of doses to the current COVID-19 vaccines is vital to boost immunity against the disease. It was stated that a single dose lowers the efficacy and plateaus the effect of the vaccine, while two doses boost its effect and efficacy [52]. In this study, the vaccines that were considered that requires two doses are Pfizer, Sinovac, AstraZeneca, and Moderna [50,51,53]. While Johnson and Johnson recommended only a single dose vaccination [54].

### 2.3 Statistical analysis

There was a total of 51 stimuli generated utilizing SPSS 25. Table 2 represents the 51 stimuli in this study which were evaluated by a 7-point Likert scale from 1 as “Strongly Disagree” to 7 as “Strongly Agree”. The orthogonal design was applied to determine the intervention of two or more variants and confirm a rational number of stimuli to be evaluated by the participants [55,56].

**Table 2:** Stimuli for conjoint analysis.

Combination	Brand	Recommendation	Cost	Efficacy	Side Effects	Vaccine Type	Doses
1	Pfizer	Personal Physician	Free from employer	50.4% Efficacy	1 in 100 patients	weakened	1 dose
2	Moderna	Personal Physician	Free from the government	66.3% Efficacy	1 in 50 patients	mRNA	1 dose
3	Sinovac	Personal Physician	Government Discount	66.3% Efficacy	1 in 2 patients	mRNA	1 dose
4	Sinovac	Personal Preference	Free from the government	95% Efficacy	1 in 2 patients	weakened	2 doses
5	Pfizer	Personal Preference	Free from the government	94% Efficacy	1 in 50 patients	mRNA	2 doses
6	Sinovac	WHO	Free from the government	95% Efficacy	1 in 2 patients	weakened	1 dose
7	Johnsons and Johnsons	Personal Preference	Fully Paid	50.4% Efficacy	1 in 2 patients	mRNA	2 doses
8	AstraZeneca	WHO	Government Discount	50.4% Efficacy	1 in 50 patients	weakened	2 doses
9	Moderna	Personal Physician	Free from the government	94% Efficacy	1 in 100 patients	mRNA	1 dose
10	Moderna	WHO	Free from employer	95% Efficacy	1 in 100 patients	weakened	2 doses
11	AstraZeneca	Personal Preference	Free from employer	66.3% Efficacy	1 in 2 patients	mRNA	1 dose
12	Johnsons and Johnsons	Personal Physician	Government Discount	95% Efficacy	1 in 50 patients	weakened	1 dose
13	Sinovac	WHO	Fully Paid	94% Efficacy	1 in 50 patients	weakened	1 dose
14	Sinovac	Personal Physician	Free from the government	50.4% Efficacy	1 in 2 patients	weakened	1 dose
15	Sinovac	Personal Preference	Government Discount	50.4% Efficacy	1 in 100 patients	mRNA	1 dose
16	Sinovac	WHO	Free from the government	50.4% Efficacy	1 in 50 patients	mRNA	2 doses
17	Johnsons and Johnsons	Personal Physician	Government Discount	50.4% Efficacy	1 in 50 patients	mRNA	2 doses
18	Pfizer	WHO	Government Discount	66.3% Efficacy	1 in 2 patients	weakened	1 dose
19	Pfizer	WHO	Free from employer	50.4% Efficacy	1 in 50 patients	mRNA	2 doses
20	Sinovac	WHO	Government Discount	94% Efficacy	1 in 2 patients	weakened	2 doses
21	Pfizer	WHO	Government Discount	76% Efficacy	1 in 2 patients	mRNA	2 doses
22	Johnsons and Johnsons	WHO	Free from the government	66.3% Efficacy	1 in 100 patients	weakened	2 doses
23	Pfizer	WHO	Fully Paid	76% Efficacy	1 in 50 patients	weakened	1 dose
24	Pfizer	Personal Physician	Free from the government	50.4% Efficacy	1 in 2 patients	weakened	1 dose
25	Johnsons and Johnsons	WHO	Free from employer	94% Efficacy	1 in 2 patients	weakened	1 dose
26	Sinovac	WHO	Government Discount	95% Efficacy	1 in 100 patients	mRNA	2 doses
27	Pfizer	Personal Physician	Fully Paid	95% Efficacy	1 in 100 patients	weakened	2 doses
28	AstraZeneca	WHO	Government Discount	95% Efficacy	1 in 100 patients	mRNA	1 dose
29	Pfizer	Personal Preference	Free from the government	95% Efficacy	1 in 50 patients	mRNA	1 dose
30	Sinovac	Personal Physician	Free from employer	76% Efficacy	1 in 2 patients	mRNA	2 doses
31	Moderna	Personal Preference	Government Discount	76% Efficacy	1 in 50 patients	weakened	1 dose
32	AstraZeneca	Personal Physician	Fully Paid	94% Efficacy	1 in 2 patients	mRNA	2 doses
33	Moderna	Personal Physician	Free from the government	66.3% Efficacy	1 in 50 patients	mRNA	2 doses
34	Sinovac	Personal Physician	Fully Paid	76% Efficacy	1 in 100 patients	mRNA	1 dose
35	Pfizer	WHO	Fully Paid	50.4% Efficacy	1 in 2 patients	mRNA	1 dose
36	Pfizer	WHO	Free from the government	95% Efficacy	1 in 2 patients	mRNA	1 dose
37	Moderna	WHO	Fully Paid	50.4% Efficacy	1 in 2 patients	mRNA	1 dose
38	Moderna	Personal Preference	Government Discount	50.4% Efficacy	1 in 2 patients	weakened	1 dose
39	Pfizer	Personal Physician	Government Discount	95% Efficacy	1 in 2 patients	mRNA	2 doses
40	AstraZeneca	Personal Physician	Fully Paid	95% Efficacy	1 in 50 patients	weakened	1 dose
41	Sinovac	WHO	Fully Paid	66.3% Efficacy	1 in 50 patients	mRNA	1 dose
42	Johnsons and Johnsons	WHO	Free from the government	76% Efficacy	1 in 100 patients	mRNA	1 dose
43	Sinovac	WHO	Fully Paid	66.3% Efficacy	1 in 2 patients	weakened	1 dose
44	Pfizer	Personal Preference	Fully Paid	66.3% Efficacy	1 in 100 patients	weakened	2 doses
45	AstraZeneca	Personal Preference	Free from the government	76% Efficacy	1 in 2 patients	weakened	2 doses
46	Sinovac	Personal Preference	Free from employer	95% Efficacy	1 in 50 patients	mRNA	1 dose
47	Moderna	WHO	Fully Paid	95% Efficacy	1 in 2 patients	mRNA	2 doses
48	Sinovac	Personal Preference	Fully Paid	50.4% Efficacy	1 in 100 patients	weakened	2 doses
49	Pfizer	Personal Preference	Government Discount	94% Efficacy	1 in 100 patients	mRNA	1 dose
50	AstraZeneca	WHO	Free from the government	50.4% Efficacy	1 in 100 patients	mRNA	1 dose
51	Johnsons and Johnsons	Personal Preference	Fully Paid	95% Efficacy	1 in 2 patients	mRNA	1 dose

## 3 Results

### 3.1 Demographics

Table 3 represents the demographics of this study. Before answering the survey, test for knowledge and awareness of the COVID-19 virus and vaccines were considered. Among the 865 participants (98.4% of the total respondents) who answered that they were knowledgeable regarding the COVID-19 vaccine, 47.9% were male and 50.5% were female. The majority of the respondents were 15–24 years old (96.8%) and are currently in the Senior High School (59.7%) and are College students (34.6%). Among the respondents, the majority had a monthly allowance/salary of less than 15,000 (85.7%) and were Roman Catholic (86.1%). The majority of the respondents are from the National Capital Region (57.5%).

**Table 3:** Demographics profile.

Characteristics	Category	N	%
Gender	Male	414	47.9
	Female	437	50.5
	Others	14	1.6
**Age**	15–24 years old	837	96.8
	25–34 years old	9	1.0
	35–44 years old	10	1.2
	45–54 years old	5	0.6
	55–64 years old	4	0.5
**Education**	Elementary graduate	8	0.90
	Junior high school graduate	516	59.7
	Senior high school graduate	299	34.6
	Technical – Vocation Graduate	2	0.20
	College Graduate	37	4.30
	Master Graduate	3	0.30
**Monthly Salary/Allowance**	Less than 15,000	741	85.7
	15,000–30,000	56	6.50
	30,000–45,000	24	2.80
	45,000–60,000	16	1.80
	60,000–75,000	8	0.90
	More than 75,000	20	2.30
**Religion**	Roman Catholic	745	86.1
	Islam	5	0.60
	Hinduism	0	0.00
	Buddhism	3	0.30
	Atheists or Agnostics	38	4.40
	Others	74	8.60
**Location**	Region I	2	0.20
	Region II	15	1.70
	Region III	9	1.00
	Region IV-A	112	12.9
	Region IV-B	162	18.7
	Region V	11	1.30
	CAR	10	1.20
	NCR	497	57.5
	Region VI	6	0.70
	Region VII	6	0.70
	Region VIII	19	2.20
	Region IX	6	0.70
	Region X	3	0.30
	Region XI	3	0.30
	Region XII	3	0.30
	Region XIII	1	0.10
	BARMM	0	0.00

### 3.2 Statistical analysis results

Presented in Table 4 and Table 5 are the utilities and average score of importance towards the different attributes and levels of preference on the COVID-19 vaccines. As seen from the results, the highest attribute considered for preference was Efficacy (40.67%), followed by Brand (24.34%), Side Effect (22.16%), and the least considered attributes were Cost (6.81%), Recommendation (3.27%), Vaccine Type (2.59%), and Doses (0.163%). With the Efficacy, the highest level considered were the highest values of 95% (0.386) and 94% (0.376). For the Brand, it was seen that Pfizer (0.22) and Moderna (0.12) were the levels preferred. The consumer also preferred the least side effect of 1 in 100 patients (0.207) or 1 in 500 patients (0.050).

**Table 4:** Utilities.

Attributes	Level	Utility Estimate	Std. Error
Brand	Pfizer	0.216	0.020
	Sinovac	−0.295	0.020
	AstraZeneca	−0.001	0.026
	Johnson and Johnson	−0.039	0.026
	Moderna	0.120	0.026
Recommendation	World Health Organization	0.032	0.015
	Personal Physician	0.004	0.017
	Personal Preference	−0.037	0.017
Cost	Free from the government	0.051	0.019
	Discounted (Government discount)	−0.047	0.019
	Fully paid	−0.073	0..019
	Free from employer	0.069	0.024
Efficacy	95%	0.386	0.020
	50.4%	−0.468	0.020
	76%	−0.049	0.026
	66.3%	−0.246	0.026
	94%	0.376	0.026
Side Effects	1 in 2 patients	−0.258	0.015
	1 in 50 patients	0.050	0.017
	1 in 100 patients	0.207	0.017
Vaccine Type	mRNA	0.027	0.011
	Weakened virus	−0.027	0.011
Doses	1	−0.002	0.011
	2	0.002	0.011
(Constant)		4.660	0.014

**Table 5:** Averaged importance score.

Importance Values	Score
Brand	24.338
Recommendation	3.2740
Cost	6.8060
Efficacy	40.672
Side Effect	22.156
Vaccine Type	2.5920
Doses	0.1630

Presented in Table 6 is the reliability of the results of this study. Based on the results, the Pearson's R correlation has a value of 0.991. This signifies that there was a very strong correlation between the attributes considered. In addition, the Kendall's Tau value of 0.909 with Kendall's Tau for Holdout with value 1.000 showed internal consistency among the responses. For which, this study considered 2 holdouts for the respondents to answer. The results showed a high level of internal validity and consistency [28,57].

**Table 6:** Correlation.

	Value	Significance
**Pearson's R**	0.991	0.000
**Kendall's Tau**	0.909	0.000
**Kendall's Tau for Holdouts**	1.000	

## 4 Discussion

Considering the rank among the attributes and levels, consumers would highly prefer the Pfizer Brand with 95% efficacy and has 1 in 100 side effects. In addition, consumers would be preferred brands recommended by the World Health Organization, free from employers, with 2 doses, and utilized mRNA vaccine type. This had a total utility estimate of 0.939. On the other hand, consumer least preferred the Sinovac Brand with 50.4% efficacy and had 1 in 2 side effects. The consumer did not consider the vaccine they need to pay for themselves, choosing by their own personal preference, 1 dose, and weakened virus vaccine type with a total utility estimate of −1.160.

From the results seen in Table 5, the highest average score of importance was the efficacy (40.67%). With the efficacy, consumers preferred 95% efficacy (0.386) followed by 94% (0.376). Consumers showed less utility estimate for 76% efficacy rate (−0.049), 66.3% (−0.246), and 50.4% (−0.468). Motta [15] stated that the Food and Drugs Administration, together with the World Health Organization presented the minimum effectiveness threshold of 50% efficacy. This information was available to the public and would therefore support why people would prefer a higher efficacy rate. Terry [58] stated that 95% efficacy of a vaccine is a significantly high rate at preventing hospitalization and death. This shows that 90% and above efficacy rate vaccines could assure consumers mild to asymptomatic symptoms on the COVID-19 virus. Bartsch et al. (2020) stated that vaccines with efficacy between 60 and 80% may still prevent severe cases of COVID-19 infection after vaccination. In addition, Katella [59] from Yale Medicine showed that the vaccine with lower than 90% efficacy rate still has a risk of being hospitalized after vaccination. With the current infectious and deadly effects of the COVID-19 virus, consumers would prefer the highest efficacy of the vaccine for self-protection.

The second highest attribute considered was the Brand (24.338%). Among the brands, the consumer would prefer Pfizer (0.216) or Moderna (0.120). AstraZeneca may also be considered (−0.001), however, Johnsons and Johnsons (−0.039) and Sinovac (−0.295) were the least preferred brands. This is consistent with the first attribute considered about the efficacy. Among the brands, Pfizer and Moderna had the highest efficacy rates of 95% and 94.7%, respectively [57,58]. In addition, Terry [59] showed that AstraZeneca had an efficacy rate of 70%, Johnsons and Johnsons with 72%, and Sinovac having 50.38%–91.25% on different clinical trials. This showed that brands were chosen based on the efficacy rates they have.

The third highest attribute considered was the side effect. Consumers preferred having the least probable side effect of 1 in 100 patients (0.207), followed by 1 in 50 patients (0.050), and least preferred 1 in 2 patients (−0.258). Riad et al. [43] showed that the side effects of vaccines are a crucial role in vaccination intention. Waters et al. [44] added that concerns with the side effects together with the perception on how it affects the person draws a negative implication towards a vaccine product [42]. Moreover, side effects are different for every individual as the body reacts differently every time. Vaccine brands or efficacy does not assure little to no side effect upon administration [60]. It was stated by Baldolli et al. [45] that proper knowledge and understanding on how the side effects affect the body is vital so people would not fear vaccination [41]. The vital role of knowledge and understanding could draw up positive interpretation among consumers with regards to vaccination [41,45].

The fourth attribute considered was the Cost which had low importance score of 6.806%. Consumers in the working sectors would prefer free vaccination from the employers (0.069) or free from the government (0.051). With consumers having to pay for the vaccine, even with discount (−0.047) or to fully pay (−0.073) were not preferred. Interestingly, the low utility scores still showed that consumers would still prefer vaccination even if they would pay for the vaccine. The significant decline of employment and other market decrease supports why consumer would prefer the COVID-19 vaccines free or with least cost [61]. Moreover, the lockdown forced the market to relieve their workers from their current position due to decrease in sales. This increased the demand for vaccination in hopes to return the normal economy status and the normal lifestyle.

The fifth attribute considered was recommendation (3.274%). The highest level among the recommendations was from the World Health Organization (0.032), followed by Personal Physicians (0.004), with Personal Preference as the least preferred (−0.037). With the results, it could be seen that people follow the recommendation stated by the World Health Organization (WHO). Kreps and Kriner [40] stated that vaccination hesitance is from consumer's perception of safety and trust among health professionals. This supports the results of this study that consumers would prefer the recommendations stated by their physician or from the WHO [41]. In addition, Kreps and Kriner [40] stated that this circumstance roots from the fact that political or organizational endorsement on vaccine acceptance have become a significant factor.

The sixth attribute was the vaccine type (2.592%). The preferred type was the mRNA (0.027) compared to the weakened virus (−0.027). In line with the highest attribute considered, Pfizer was preferred. Pfizer is a vaccine brand that utilizes mRNA vaccine type, as well as Moderna [58]. This provides further consistency proof with regards to the results of this study. Other brands utilized weakened or adenovirus-based vaccine types, which is consistent with the result of this study as the least preferred type of COVID-19 vaccine. Both vaccine types are similar in a way that it introduces the COVID-19 virus to create antibodies [59]. However, the mRNA virus is introduced in the body as a piece of genetic code to enable bodies to create blueprints to fight off the actual virus. The blueprints introduced to the body creates a spike protein, so the COVID-19 virus does not penetrate the cells of the body if ever infected. For the weakened virus, the introduction of the virus to the body forces the body to create antibodies to fight off the foreign object administered [59]. This provides further information as to why consumers prefer mRNA over the actual virus administration from the COVID-19 vaccine.

Lastly, the last attribute considered for preference was the dose (0.163%). The consumer would prefer 2 doses (0.002) rather than a single dose (−0.002). Polack et al. [62] concluded that a two-dose vaccine showed 95% protection. This supports the result of this study on why consumers would prefer the two-dose vaccine rather than a single-dose vaccine. Moreover, among the different brands of COVID-19 vaccines available, only Johnson and Johnson have a single-dose vaccination type while other brands require two doses [59]. This further shows overall consistency among the responses of the consumers.

The results of this study provided preference of consumers highlighting safety due to the brand and efficacy rate as the main preference. As supported by Motta [15] and Baldolli et al. [45], consumer prefer their safety as the main priority when it comes to vaccination [15,45]. Consumers do proper research and gather information with regards to choosing vaccines. In line with the findings of Riad et al. [43] and Waters et al. [44], consumers would prefer those of little to no effects on the body. Furthermore, the knowledge and understanding of the different vaccines enabled the preference among all vaccines indicated. The knowledge obtained from the World Health Organization would play a crucial role in preference among the vaccine brands. Moreover, Zandian et al. [41] discussed how the students would rely more on the health benefit declaration of reliable sources such as the Ministry of Health or the World Health Organization [40,41].

### 4.1 Practical contribution

The results of this study showed that the younger generation would prefer the highest efficacy rate among all brands of COVID-19 vaccine as expected. However, with the lack of supply, Zandian et al. [41] discussed how proper knowledge dissemination among students would help promote vaccine uptake whatever the brand may be. This finding may be utilized to promote herd vaccination. As stated in different studies, herd vaccination would lead to community protection against the COVID-19 virus; which is the ultimate goal of vaccination against COVID-19 [7,[14], [15], [16]]. Moreover, the results of this study could be utilized in other vaccination. The more efficient the vaccine, the more people would accept the vaccine. In accordance with the public's preference, the acceptance of vaccination may be promoted to have most of the people vaccinated. With proper knowledge and understanding, people would accept vaccination. Lastly, as stated by Wong et al. [16], the government's recommendation is a significant driving factor towards the acceptance of the COVID-19 vaccine. The results of this study could be utilized by the government to further promote vaccination considering the preference of the younger community.

### 4.2 Limitations

This study considers different limitations. First, this study was conducted during the COVID-19 pandemic. The results of the study have considered the high level of fear among the different respondents due to the current steady increase of the COVID-19 infection, especially in the Philippines. This led to the result of consumers considering the highest efficacy rate possible. Second, this study considered only the top current brand in the market such as Pfizer, Modera, Johnson and Johnson, Sinovac, and AstraZeneca. There are other brands available such as Sputnik V, Novavax, CanSino Biologic, and Bharat Biotech that were not considered may result to different importance in the levels of brands. Lastly, this study only focused on the preference among the different COVID-19 vaccines for mostly 15 years old and above since they are the ones next in line to uptake the COVID-19 vaccines. In addition, it is suggested to consider the combination of younger and older generation to be able to conclude a generalized result. To which, a different importance of attribute and its level may be obtained and discussed. Moreover, it is recommended to integrate this study with the intention to have the COVID-19 vaccine such as utilizing the Structural Equation Modeling or even utilize artificial intelligence in trying to analyze ways to combat the COVID-19 virus similar to the study of Tkatek et al. [63]. Lastly, an extension using clustering technique may also be applied to highlight sectors and demographic attributes influencing vaccine uptake using machine learning algorithm [[64], [65], [66]].

## 5 Conclusion

Vaccines are utilized to prevent the severity of illnesses like the COVID-19 virus [67,68]. This research aimed to analyze the preference on the existing vaccine attributes of COVID-19. Specifically, this study considered 7 attributes such as cost, brand, recommendations, efficacy, side effects, vaccine type, and dose. This study utilized conjoint analysis with orthogonal design among 865 respondents. There was a total of 51 stimuli created for a self-administered questionnaire utilizing a 7-point Likert scale.

The result showed that consumers considered brand as the highest attribute, specifically Pfizer and Moderna among other brands. Moreover, the efficacy of 90% and higher were the preferred vaccine with 1 in 100 patient side effects reported. It was seen that safety and effectiveness are the priority in choosing a COVID-19 vaccine. The findings of this study showed that knowledge and understanding of the COVID-19 vaccine would drive consumer's preference for the vaccines available. The results of this study would help in marketing the COVID-19 vaccine among younger generations. Moreover, the findings of this study could be utilized by the government to increase the willingness to be vaccinated and eventually achieve herd immunity.

## Funding

This research was funded by Mapúa University Directed Research for Innovation and Value Enhancement (DRIVE).

## Institutional review board statement

This study was approved by Mapua University Research Ethics Committees and followed the National Ethical Guidelines for Health and Health-Related Research 2017 by the Philippine Health Research Ethics board.

## Informed consent statement

Informed consent was obtained from all subjects involved in the study.

## Data availability

Data available upon request.

## Declaration of competing interest

The authors declare that they have no known competing financial interests or personal relationships that could have appeared to influence the work reported in this paper.
